# Beyond CHNAS: Performance Measurement for Community Health Improvement

**DOI:** 10.5334/egems.312

**Published:** 2019-08-20

**Authors:** Michael A. Stoto, Mary V. Davis, Abby Atkins

**Affiliations:** 1 Georgetown University, US; 2 Health Resources in Action, US

**Keywords:** Community Health Needs Assessment (CHNA), Community health improvement process, Shared measurement systems, Population health data, Performance measurement

## Abstract

**Research Objective:**

Non-profit hospitals are required to work with community organizations to prepare Community Health Needs Assessment (CHNA) and implementation strategy (IS). In concert with the health care delivery system’s transformation from volume to value and efforts to enhance multi-sector collaboration, such community health improvement (CHI) processes have the potential to bridge efforts of the health care delivery sector, public health agencies, and community organizations to improve population health. Having a shared measurement system is critical to achieving collective impact, yet despite the availability of community-level data from a variety of sources, many CHI processes lack clear, measurable objectives and evaluation plans. Through an in-depth analysis of ten exemplary CHI processes, we sought to identify best practices for population health measurement with a focus on monitoring collaborative implementation strategies.

**Study Design:**

Based on a review of the scientific literature, professional publications and presentations, and nominations from a national advisory panel, we identified 10 exemplary CHI processes. Criteria of choice were whether (1) the CHIs articulate a clear definition of intended outcomes; (2) clear, focused, measurable objectives and expected outcomes, including health equity; (3) expected outcomes are realistic and addressed with specific action plans; and (4) whether the plans and their associated performance measures become fully integrated into agencies and become a way of being for the agencies. We then conducted an in-depth analysis of CHNA, IS, and related documents created by health departments and leading hospitals in each process.

**Population Studied:**

U.S. hospitals.

**Principal Findings:**

Community health improvement processes benefit from a shared measurement system that indicate accountability for specific activities. Despite the importance of measurement and evaluation, existing community health improvement efforts often fall short in these areas. There is more variability in format and content of ISs than CHNAs; the most developed models include population-level goals/objectives and strategies with clear accountability and metrics. Other hospital IS’s are less developed.

Although all U.S. hospitals are familiar with performance measurement in their management, this familiarity does not seem to carry over to Community Benefit and CHNA efforts. Indeed, 5 of the 10 CHI processes we examined have some Accountable Care Organization (ACO) involvement, where population-health performance measures are commonplace. Yet this involvement is not mentioned in the CHNAs and ISs, nor are ACO data cited.

**Conclusions:**

Strengthening the CHNA regulations to require that hospitals report the evaluation measures they intend to monitor based on an established community health improvement model could help communities demonstrate impact. As in other areas of health care, performance measures should be tailored to implementation strategy, with clear indication of accountability, and move from outputs to process and outcome measures with established validity and reliability.

**Implications for Policy or Practice:**

Although performance measurement is now commonplace throughout the health care system, the individuals who manage CHI processes may not be that familiar with this approach. This suggests that it is important to develop practitioners’ knowledge and skills needed to use it population health data effectively.

## Introduction

Since the passage of the *Affordable Care Act* (ACA), all non-profit hospitals are required to conduct a Community Health Need Assessment (CHNA) at least every three years and adopt an implementation strategy (IS) describing how identified needs will be addressed. As we describe in more detail in the Background section, taken together with other provisions in the ACA intended to encourage the health care delivery sector to be accountable for health outcomes in defined populations, as well as the increasing prominence of performance management in the health care delivery sector, the new CHNA requirements – and especially their implementation strategies – have the potential to refocus health care providers’ attention “upstream” to address the social and behavioral determinants of health. Such community health improvement (CHI) processes offer an extraordinary *promise* for advancing population health through multi-sector collaboration to build health partnerships [1]. However, although there are a number of promising models being developed by specific hospitals and communities, evaluations of current efforts suggest that the new IRS regulations [2] do not yet seem to have not achieved their potential in most communities.

This analysis is part of a larger project aimed at increasing our understanding of the characteristics of CHI processes, employed by hospitals and their community partners, that have promise to improve health equity and outcomes. An environmental scan we conducted for this project [3] reviewed assessments of recent CHNAs and ISs. Recognizing that some of these related to the first round of hospital CHNAs, so are possibly out of date, one of the consistent findings was that there was far more focus on conducting CHNAs than on implementing strategies, monitoring these efforts, and evaluating the results. The literature tells us that a “shared measurement system” is critical to achieving collective impact [4], yet despite the availability of community-level data from a variety of sources, many current CHI processes seem to lack clear, measurable objectives and evaluation plans.

To follow this lead, we conducted an in-depth analysis of ten exemplary CHI processes to identify best practices. We take as our starting point that population health is a shared responsibility, and that managing it requires two different measurement strategies. First, there should be a shared community health profile to identify common priorities. Second, implementation plans for hospitals and other entities should include pre-specified performance measures to align efforts and to ensure accountability for the organizations’ actions to address community health improvement [5,6,7]. A companion paper [8] addresses measures for community health needs assessments and priority setting. This paper sets the stage and describes our methods, and focuses on current practices in performance measures associated with CHI processes Implementation Strategies.

## Background

The U.S. health care delivery system is in a transition in focus from clinical care to population health. This transition is taking place because the current system – in which health care providers are reimbursed based on the volume of services they provide – is unsustainable. This transition has spawned payment models that reward value – including improvements in population health – and move away from fee-for-service models that reward volume such as Accountable Care Organizations (ACO), Accountable Health Communities (ACH), Community-Centered Health Homes (CCHH), and patient centered medical homes (PCMH). The principles and specific initiatives mandated by the ACA, particularly the Centers for Medicare and Medicaid Services’ (CMS) State Innovation Model (SIM) initiative, are important factors in this transformation. As a result, health care delivery systems are increasingly being held accountable for improving health outcomes, which they realize they cannot do without collaboration with other entities in the communities they serve.

Just as the health care sector has expanded its focus beyond illness treatment alone to the “social determinants of health” – the complex set of forces and systems shaping the conditions of daily life that drive health outcomes, such as inequality, social mobility, community stability, and the quality of civic life [9], DeSalvo and others [10] have declared the need to re-envision public health practice in the United States along the same lines. This concept can be seen in many efforts aimed at multi-sector collaboration in public health, health care delivery organizations, community-based organizations, and the private sector. These include such varied activities as health department accreditation [11], the Robert Wood Johnson Foundation’s Culture of Health initiative [1], the Health in All Policies (HiAP) approach [12], and the San Francisco Federal Reserve Bank’s Pathways to System Change initiative [13]. Despite their varied rationale and organizational forms, the key elements of these initiatives are well described by Kania and Kramer’s Collective Impact framework [4], which includes: 1) a common agenda, 2) shared measurement systems, 3) mutually reinforcing activities, continuous communication, and a backbone support organization.

Our environmental scan [3] also identified many federal and state regulations, guidance documents, community health improvement models, related initiatives, as well as sources of community health data and other resources. This includes the Internal Revenue Service’s final §501(r) regulations regarding CHNAs [2] as well as many state regulations and expectations regarding community benefits, hospital and or local health department community health assessments and implementation strategies. The IRS community benefit and CHNA regulations have spawned or enhanced the development of a plethora of guidance documents, CHNA models, data systems, and other resources. These include comprehensive hospital-focused models such as *Assessing & Addressing Community Health Needs* [14] and the *Community Health Assessment Toolkit* [15]. Other resources, include state community health improvement models and data systems, the County Health Rankings & Roadmaps [16], the CHNA.org toolkit [17], the Center for Disease Control and Prevention’s (CDC) Community Health Improvement Navigator [18], and the de Beumont Foundation’s Practical Playbook [19].

## Methods

**Figure 1:**
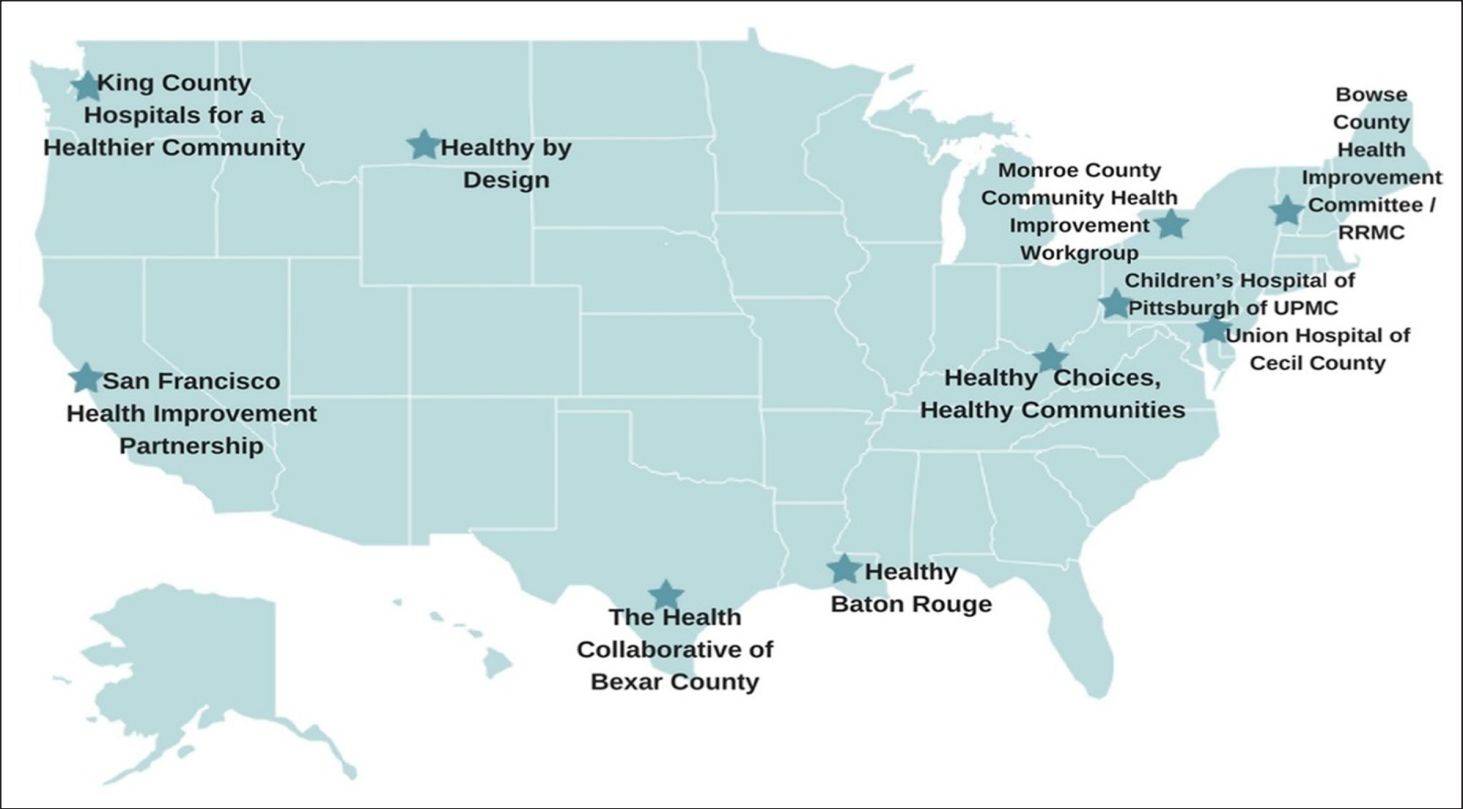
Location of study sites. Source: Atkins et al., [20].

This analysis is based on an in-depth analysis of 10 exemplary CHI processes identified based on a review of the scientific literature, professional publications and presentations, and nominations from a national advisory panel. The selection criteria were whether (1) the CHIs articulate a clear definition of intended outcomes; (2) clear, focused, measurable objectives and expected outcomes, including health equity; (3) expected outcomes are realistic and addressed with specific action plans; and (4) whether the plans and their associated performance measures become fully integrated into agencies and become a way of being for the agencies [20]. The communities studied are displayed in Figure 1. For each community we conducted an in-depth analysis of CHNAs, ISs, and related documents created by health departments and hospitals, and this was supplemented by a 2- to 3-day site visit.

## Findings

Compared to the CHAs/CHNAs analyzed in the companion paper, the implementation strategies in the CHI processes we reviewed generally displayed inconsistent or minimal alignment among collaborative members [8]. This is consistent with the findings of the environmental scan, that the implementation phase of community health improvement processes tends to be less developed than the assessment and priority setting phases [3].

The format and content of ISs in the 10 CHI processes we reviewed also varied more than the CHNAs. The most developed ISs included specific strategies, activities, and so on, with clear accountability and metrics. For example, one strategy in Methodist Hospital’s (Bexar County) diabetes priority is to provide diabetes education to patients. This was accompanied with a specific performance measure (the number of classes and individual instruction provided to patients) and target (Methodist Hospital anticipates at least 1,500 diabetes educator visits in 2017). Another strategy is to provide volunteer staff for a health fair in a lower socioeconomic neighborhood to provide screenings for glucose, blood pressure, full lipid panel cholesterol and BMI checks, as well as literature. For this strategy, the performance measure is the number of individuals screened, and the target is 150 participants.

Similarly, Ashland King’s Daughter Medical Center’s obesity priority has the goal of improving patient knowledge about the relationship between height and weight through screening, counseling and education in health care settings. One strategy is to provide weight and BMI screening during office visits. The associated performance measure is the number of patient contacts that include assessment of BMI. The target is to increase the number by 2 percent annually 2018–2019, compared to a benchmark to be established in 2017.

Another example relates to the East Baton Rouge parish priority to reduce childhood obesity through education. This strategy includes as an Action step: Use the “5210 + 10” message (a national program encouraging 5 servings of fruits and vegetables each day, 2 hours or less of recreational screen time per day, 1 hour of physical activity per day, 0 sweetened or sugary drinks, and 10 hours of sleep every night for children) in all primary care clinics health care settings. The performance measure is the number of primary care clinics using this message, and the target for Baton Rouge General Hospital is 15.

In some cases, community health improvement plans (CHIPs) or implementation strategies listed what might be called “first step” measures. For instance, the Ashland-Boyd CHIP included under Goal #1 (Increase active membership in the Healthy Choices Healthy Communities coalition serving Boyd, Carter, Greenup and Lawrence counties) three “Objectives”

- Aggregate list of potential new coalition partners by September 2016 and issue invitations to join Healthy Choices Healthy Communities coalition, by December 2016.
- Create a calendar and notification strategy of quarterly full Healthy Choices Healthy Communities coalition meetings, by September 2016.
- Grow each chartered workgroup by a minimum of three new members, by December 2016.

Other CHI processes we reviewed listed population-level goals and/or objectives but not specific performance measures. Cecil County’s chronic disease priority, for instance, tracks high blood pressure among adults using Maryland BRFSS and State Health Improvement Plan (SHIP) measures. The Union Hospital/Cecil County Health Department joint CHIP includes the specific target: By June 30, 2019, reduce high blood pressure among adults by 5 percent; baseline: 30.1 percent in 2006–2012.

Similarly, the Monroe County Heart Health Management and Prevention priority monitors the percentage of adults ages 18 or older with hypertension who have controlled their blood pressure using data from a local high blood pressure registry. The Monroe County CHIP, which applies to the four hospitals in the county, includes the target: Increase the percentage of adults ages 18+ with hypertension (in the registry) who have controlled their blood pressure from 68.9 percent to 71.5 percent.

When they are included in published implementation strategies with clearly identified accountability, the performance measures tend to be outputs, such as attendance at events, seminars, classes and screenings, as in the Ashland-Boyd and Baton Rouge examples. Baseline data, measure specifications, and time-limited targets, however, are often lacking. Health outcomes, such as in the Cecil and Monroe county examples, are uncommon. Arguably, measures like these are not effective performance measures since they are not tailored to specific strategies and accountability is not clearly indicated. For example, if the prevalence of high blood pressure does not go down as anticipated, it is not clear whether the hospital, health department, or a combination of partners should be held accountable.

Kaiser Permanente IS’s do not list specific strategies or activities, but rather anticipate contracting with community-based organizations using Community Benefit funds and evaluating the results. Kaiser Foundation Hospital (KFH) San Francisco’s evaluation Plans are worth considering as a model. They state:

KFH San Francisco will monitor and evaluate the strategies listed above for the purpose of tracking the implementation of those strategies as well as to document the anticipated impact. Plans to monitor will be tailored to each strategy and will include the collection and documentation of tracking measures, such as the number of grants made, number of dollars spent, and number of people reached/served. In addition, KFH San Francisco will require grantees to propose, track and report outcomes, including behavior and health outcomes as appropriate. For example, outcome measures for a strategy that addresses obesity/overweight by increasing access to physical activity and healthy eating options might include number of students walking or biking to school, access to fresh locally grown fruits and vegetables at schools, or number of weekly physical activity minutes [21].

Beyond these examples, the implementation strategies of other hospitals in the CHI processes we studies are less developed, especially from a performance measurement point of view. For example, Children’s Hospital of Pittsburgh of UPMC’s CHNA & IS report includes a detailed implementation plan with specific programs, anticipated impacts, and planned collaborations. To address childhood obesity, for instance, the “Obesity Prevention Initiatives through Early Care and Family Support Centers” (program) has a year 3 goal (anticipated impact) to “improve quality of food served at family support and early child care environments” and 27 health sector, public health, academic, and community-based organizations are listed under “planned collaborations.” There are, however, no quantitative or qualitative performance measures for this or other strategies listed in the report. Other hospitals, for example Ashland Bon Secours Our Lady of Bellefonte Hospital, does not publish its implementation strategy on the hospital website.

## Discussion

**Figure 2:**
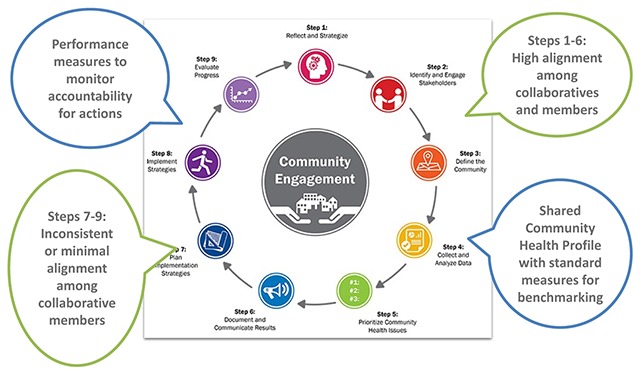
ACHI Community Health Improvement Model. Source: Adapted from Association for Community Health Improvement [15].

As noted above, best practices in community health improvement require two separate types of measures [7,5,6]. In order to identify community health needs and set priorities, data are needed describing both the community’s health outcomes as well as the factors, including upstream, that affect health outcomes. This is represented as steps 3, 4 & 5 in ACHI Community Health Improvement Model (Figure 2), and this need is fulfilled by the types of CHNA data discussed in the companion paper [8]. Once priorities are identified, and implementation strategies are developed, performance measures are needed to track implementation progress and hold collaborators accountable for the actions they take towards common goals. This corresponds to steps 7, 8 & 9 in ACHI Community Health Improvement Model.

To be most effective in aligning and managing coalition partners’ activities, performance measures appropriate to the nature of the programs and activities in the implementation strategy are needed. This includes operational measures directly associated with the programs and activities in the implementation strategy, such as the number of influenza vaccines delivered to high risk patients or the number of people who attended weight loss sessions. It also includes outcome measures, health status indicators expected to be impacted by specific programs and activities in the implementation strategy, such as a reduction in the incidence of influenza in the high-risk population and weight loss in the targeted population. As in other areas of health care, these measures should be tailored to implementation strategy, with clear indication of accountability [22].

The final IRS regulations include a requirement that hospitals’ CHNA reports include an evaluation of the impact of any actions that were taken since the hospital facility finished conducting its immediately preceding CHNA to address the significant health needs identified in the hospital facility’s prior CHNA(s) [2]. Hospitals may see this as an opportunity to evaluate the impact of their activities. But a CHNA is a needs assessment, not an intervention or implementation strategy. Furthermore, population health outcomes are influenced by many factors other than those in the Implementation Strategy, and even if successful, it may take many years for a change in outcomes to be seen. Because of this, although the ultimate goal is improvement in population health outcomes, performance measurement in Implementation Strategies should include process measures for two reasons. From a program management point of view, as long as the implementation strategy’s programs and activities are evidence based, we can assume that outcomes will follow. From an evaluation perspective, process measures are needed to demonstrate that the population received the interventions as planned to help build the case that improved health outcomes could be attributed to the interventions.

## Conclusions

As noted by Kania and Kramer, agreement on a common agenda is illusory without agreement on the ways success will be measured and reported [4]. Community health improvement processes benefit from a shared measurement system that indicate accountability for specific activities. Despite the importance of measurement and evaluation, existing community health improvement efforts often fall short in these areas. Strengthening the CHNA regulations to require that hospitals report the evaluation measures they intend to monitor based on an established community health improvement model could help communities demonstrate impact [23].

A recent study conducted for the Collective Impact Forum by ORS Impact and Spark Policy Institute [24] examined 25 collective impact initiatives across the U.S. with the goal of surfacing insights about when and how collective impact achieves impact. One of the common challenges was the relative lack of “nearer-term measures”:

Sites that only included long-term outcomes in their shared measurement system experienced significant challenges around data use. For these initiatives, the shared measurement system was primarily focused on measuring progress/accountability as opposed to generating data that could be used to inform policy or refine strategy implementation. Shorter-term metrics were needed to help inform the day-to day work of the initiative. In the context of CHI processes, this problem can be translated to the need for performance measures associated with the collaboration’s implementation strategy, not just the outcome measures needed to set priorities in the CHNA [24].

By this standard, most of the 10 CHI processes that we examined in our case studies have opportunities to improve how they use performance measurement to advance their community health goals. Ironically, although all U.S. hospitals are familiar with performance measurement in their management, this familiarity does not seem to carry over to Community Benefit and CHNA efforts. Indeed, 5 of the 10 CHI processes we examined [20] have some ACO involvement, and one was part of an Accountable Health Community (AHCs), where population-health performance measures are commonplace. Yet this involvement is not mentioned in the CHNAs and ISs, nor are ACO or AHC data cited.

Although performance measurement is now commonplace throughout the health care system, the individuals who manage CHI processes may not be that familiar with this approach. This suggests that it is important to develop practitioners’ knowledge and skills needed to use it population health data effectively. Here we can build on the growing effective data use in value-based purchasing arrangements such as ACOs and AHCs.

## References

[1] Plough, AL. Building a culture of health: challenges for the public health workforce. American Journal of Preventive Medicine. 2014; 47(5 Supp 3): S388–S390. DOI: 10.1016/j.amepre.2014.07.037 25439263

[2] Internal Revenue Service. Additional rules for charitable hospitals, 79 Fed. Reg. 78953. 2014.

[3] Stoto, MA, Klaiman, T and Davis, MV. A community health needs assessment environmental scan. 10 13, 2018 [cited 2018 Oct 28]. Available at SSRN: https://papers.ssrn.com/sol3/papers.cfm?abstract_id=3230259 DOI: 10.2139/ssrn.3230259

[4] Kania, J and Kramer, M. Collective impact. Stanford Social Innovation Review. 1 2011; 9(1): 36–41.

[5] Institute of Medicine. Improving health in the community: a role for performance monitoring Washington, DC: National Academy Press; 1997. 25121202

[6] Institute of Medicine. For the public’s health: the role of measurement in action and accountability Washington, DC: National Academies Press; 2010.

[7] Stoto, MA and Ryan-Smith, C. Community Health Needs Assessments: aligning the Interests of Public Health and the Healthcare Delivery System to Improve Population Health. National Academy of Medicine Discussion Paper. 2015 [cited 2018 Oct 28]. Available from: http://nam.edu/perspectives-2015-community-health-needs-assessments-aligning-the-interests-of-public-health-and-the-health-care-delivery-system-to-improve-population-health/ DOI: 10.31478/201504h

[8] Stoto, MA, Davis, MV and Atkins, A. Making Better Use of Population Health Data for Community Health Needs Assessments. eGEMs. 2019; 7(1): 44, pp. 1–9. DOI: 10.5334/egems.305 PMC6706997 31497616

[9] Norris, T and Howard, T. Can Hospitals Heal America’s Communities? “All in for Mission” is the Emerging Model for Impact, Democracy Collaborative. 2015 [cited 2018 Oct 28]. Available from: http://democracycollaborative.org/content/can-hospitals-heal-americas-communities-0.

[10] DeSalvo, KP, O’Carroll, PW, Koo, D, Auerbach, JM and Monroe, JA. Public health 3.0: time for an upgrade. American Journal of Public Health. 2016; 106(4): 621–622. DOI: 10.2105/AJPH.2016.303063 26959263 PMC4816012

[11] Laymon, B, Shah, G, Leep, CJ, Elligers, JJ and Kumar, V. The proof’s in the partnerships: are affordable care act and local health department accreditation practices influencing collaborative partnerships in community health assessment and improvement planning? Journal of Public Health Management and Practice. 2015; 21(1): 12–17. DOI: 10.1097/PHH.0000000000000087 25414951

[12] Health in All Policies (HiAP) Steering Committee. Health in all policies: strategies to promote innovative leadership. Association of State and Territorial Health Officials. 1, 2013; 2016 [cited 2018 Oct 28]. Available from: http://www.astho.org/Programs/Prevention/Implementing-the-National-Prevention-Strategy/HiAP-Toolkit/.

[13] Siegel, B, et al. Pathways to system change: the design of multisite, cross-sector initiatives. Federal Reserve Bank of San Francisco, Working Paper 2015–03. 7 21, 2015 [cited 2018 Oct 28]. Available from: https://www.frbsf.org/community-development/publications/working-papers/2015/july/pathways-to-system-change-multisite-cross-sector-initiatives/.

[14] Catholic Health Association of the United States. Assessing and Addressing Community Health Needs. 2015 [cited 2018 Oct 28]. Available from: https://www.chausa.org/communitybenefit/assessing-and-addressing-community-health-needs.

[15] Association for Community Health Improvement. Community Health Assessment Toolkit. 2017 [cited 2018 Oct 28]. Available from: www.healthycommunities.org/assesstoolkit.

[16] University of Wisconsin Population Health Institute. County health rankings and roadmaps. 2014 [cited 2018 Oct 28]. Available from: http://www.countyhealthrankings.org/.

[17] Community Commons. Community Health Needs Assessment. 2016 [cited 2018 Oct 28]. Available from: http://www.communitycommons.org/chna/.

[18] Centers for Disease Control and Prevention. Community Health Improvement Navigator. 2016 [cited 2018 Oct 28]. Available from: http://www.cdc.gov/chinav/index.html.

[19] Practical Playbook. 2016 [cited 2018 Oct 28]. Available from: https://www.practicalplaybook.org/.

[20] Davis, MV, Atkins, A, Mikolowsky, K, Guptil, M and Stoto, MA. CHI Processes Evaluation Evaluating the Promise of Community Health Improvement Processes Prepared for the Robert Wood Johnson Foundation, Health Resources in Action, January, 2019 [cited 2019 July 22]. Available from: https://hria.org/wp-content/uploads/2019/01/CHI-Processes-Eval-Final-Report-013119.pdf.

[21] Kaiser Foundation Hospital-San Francisco. Implementation Strategy Report for Community Health Needs. 2017; 2016 [cited 2018 Oct 28]. Available from: https://share.kaiserpermanente.org/wp-content/uploads/2013/10/KFH-San-Francisco-IS-Report.pdf.

[22] Massachusetts Attorney General’s Office. The Attorney General’s Community Benefits Guidelines for Non-Profit Hospitals. 2018 [cited 2018 Oct 28]. Available from: https://www.mass.gov/service-details/community-benefits-guidelines.

[23] Stoto, MA and Davis, MV. Achieving the Promise of Community Health Needs Assessments. Academy Health Blog. 6, 2019 [cited 2019 July 22]. Available from: https://www.academyhealth.org/blog/2019-06/achieving-promise-community-health-needs-assessments.

[24] ORS Impact. When Collective Impact has an Impact: A Cross-site Study of 25 Collective Impact Initiatives. 2018 [cited 2018 Oct 28]. Available from: http://orsimpact.com/blog/When-Collective-Impact-Has-Impact-A-Cross-Site-Study-of-25-Collective-Impact-Initiatives.htm.

